# Supplementary material for: Splice-Junction-Based Mapping of Alternative Isoforms in the Human Proteome
Source: Cell Rep. Author manuscript; Available in PMC 2020 Jan 15. (PMC6961840; doi:10.1016/j.celrep.2019.11.026)
Supplement: 2 [file NIHMS1546469-supplement-2.zip › DF1/PXD006675/CardiacFibroblast/CardiacFibroblast_7_STK39_EENPEGPPNANEDYR.pdf]

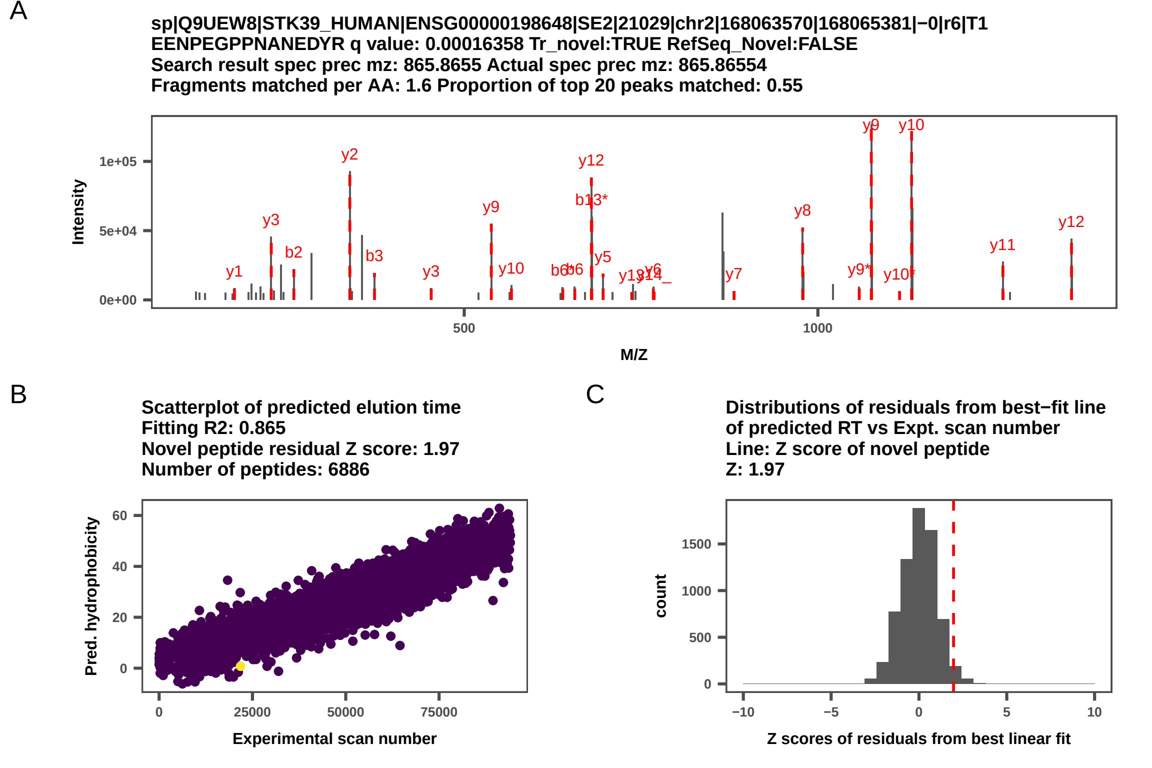
A
sp|Q9UEW8|STK39_HUMAN|ENSG00000198648|SE2|21029|chr2|168063570|168065381|−0|r6|T1
EENPEGPPNANEDYR q value: 0.00016358 Tr_novel:TRUE RefSeq_Novel:FALSE
Search result spec prec mz: 865.8655 Actual spec prec mz: 865.86554
Fragments matched per AA: 1.6 Proportion of top 20 peaks matched: 0.55
Intensity
1e+05
5e+04
0e+00
500
1000
M/Z
y1
y3
b2
y2
b3
y3
y9
y10
b6
y12
b13*
y5
y7
y8
y9*
y9
y10*
y10
y11
y12
B
Scatterplot of predicted elution time
Fitting R2: 0.865
Novel peptide residual Z score: 1.97
Number of peptides: 6886
Pred. hydrophobicity
60
40
20
0
0
25000
50000
75000
Experimental scan number
C
Distributions of residuals from best−fit line
of predicted RT vs Expt. scan number
Line: Z score of novel peptide
Z: 1.97
count
1500
1000
500
0
−10
−5
0
5
10
Z scores of residuals from best linear fit
